# Antioxidant and antiproliferative potentials of phenolic-rich extracts from biotransformed grape pomace in colorectal Cancer

**DOI:** 10.1186/s12906-023-03852-w

**Published:** 2023-02-01

**Authors:** Katarina Mišković Špoljarić, Gordana Šelo, Ena Pešut, Josipa Martinović, Mirela Planinić, Marina Tišma, Ana Bucić-Kojić

**Affiliations:** 1 Josip Juraj Strossmayer University of Osijek, Faculty of Medicine, Josipa Hutlera 4, 31000 Osijek, Croatia; 2 Josip Juraj Strossmayer University of Osijek, Faculty of Food Technology Osijek, Franje Kuhača 18, 31000 Osijek, Croatia

**Keywords:** Wine production residues, Phenolic compounds, Solid-state fermentation, Bioactivity, Colorectal cancer cell lines

## Abstract

**Background:**

Colorectal carcinoma is one of the most commonly diagnosed malignancies worldwide. Consumption of dietary supplements and nutraceuticals such as phenolic compounds may help combat colorectal carcinoma. The effect of two phenolic-rich extracts prepared from biotransformed grape pomace on the antioxidant properties and antiproliferative activity against two colorectal cancer cell lines (Caco-2 and SW620) were investigated.

**Methods:**

A 15-day solid-state fermentation with the white-rot fungi *Phanerochaete chrysosporium* and *Trametes gibbosa* was used to biotransform grape pomace. Solid-liquid extraction was then performed to extract bioactive compounds. The extract was analyzed for the determination of phenolic compounds by ultra-high performance liquid chromatography and in vitro assays of biological activities (antioxidant activity, antiproliferative activity, cell cycle analysis).

**Results:**

The 4 days of solid-state fermentation proved to be the optimal period to obtain the maximum yield of phenolic compounds. The tested extracts showed significant antioxidant and antiproliferative activities. Grape pomace treated with *P. chrysosporium* and *T. gibbosa* reduced cancer cell growth by more than 60% at concentrations (solid/liquid ratio) of 1.75 mg/mL and of 2.5 mg/mL, respectively. The cell cycle perturbations induced by the grape pomace extracts resulted in a significant increase in the number of cells in the S (9.8%) and G2/M (6.8%) phases of SW620 exposed to *T. gibbosa* after 48 hours, while *P. chrysosporium* increased the percentage of cells in the G1 phase by 7.7%. The effect of grape pomace extracts on Caco-2 was less pronounced.

**Conclusions:**

The obtained results suggest the presence of bioactive compounds in biotransformed grape pomace as a residue from winemaking, which could be used to prevent colon cancer.

## Introduction

Colorectal carcinoma is the second deadliest and third most commonly diagnosed malignancy [1, 2] with over 1.9 million new cases (10% of all new cases regardless of gender and age) and more than 935,000 deaths in 2018 according to Bray et al. [3]. Although colorectal cancer has multifactorial causes, most of them are related to poor dietary habits. Among dietary supplements, phenolic compounds represent the most interesting and well-studied class of compounds that can be used as therapeutics for a variety of common diseases, including cancer [4]. In recent years, numerous studies [5–7] have shown a protective effect of plant phenolic compounds against colorectal carcinoma. The use of agro-food industry side streams to produce a wide range of value-added products, including phenolic compounds [8–11], is one of the key strategies of the biorefinery paradigm towards a sustainable circular bioeconomy. In this context, the cascade use of certain biomass is of great importance, as in this way several different products can be produced from the same waste feedstock within the same or different biorefineries [12]. Emphasis is placed on the introduction of environmentally friendly and cost-effective technologies to achieve zero-waste production, and on the development and commercialization of novel biobased formulations [13].

Grape pomace (GP) is the residue generated during wine production and consist mainly of the skin, pulp, seeds, and stems. It is a source of bioactive phenolic compounds, including flavonoids (flavan-3-ols, anthocyanins, and flavanols) and non-flavonoids (phenolic acids), which are being studied in the context of preventive measures against cardiovascular diseases and tumours, and in the treatment of nervous and brain disorders and arthritis [14, 15]. Due to the antioxidant and anti-inflammatory properties of these compounds, the extracts of GP may have a number of beneficial effects in health care [16–18]. Flavonoids have various anticancer effects, including introduction of apoptosis, cell cycle arrest, antiproliferative, antioxidative, antiangiogenic, and anti-metastatic activities against many human cancer cell lines [4]. Previous research suggests that grape seed extracts have a preventive effect on colorectal carcinoma due to their phenolic compounds and antioxidant activity, as they can scavenge reactive oxygen and nitrogen species, thereby reducing oxidative stress, increasing epithelial barrier integrity, and reducing inflammation in the gut [19, 20]. In addition, extracts of GP and/or grape stems may be effective in preventing certain cancers (prostate, leukaemia, breast, skin, and colon) through mechanisms such of inhibition of cell proliferation, cell cycle arrest, induction of apoptosis, and interruption of intracellular signal transduction [17, 21]. It is well known that the functionality of phenolic compounds is limited by the fact that many of them, such as polymers, glycosides and esters, are not always bioavailable. Enzymatic hydrolysis and bioprocesses such as fermentation can result in the release of phenolic components from their conjugates, leading to increased bioactivity [22]. The bioprocess that has been shown to be effective in releasing large amounts of phenolic compounds and increasing their bioactivity is solid-state fermentation (SSF) [23]. Due to its economic and sustainable properties, it has been widely explored and applied to obtain high-value compounds from organic wastes [24]. Usually, white-rot fungi are used in SSF, which produce a complex system of lignolytic enzymes during their growth, in which enzymatic hydrolysis occurs, free phenolic compounds are released, and those with lower molecular weight are formed, which contribute to the enhancement of antioxidant activity [24–26]. In this study, the antioxidant properties and antiproliferative properties of extracts from biologically treated GP were investigated on two human colon carcinoma cell lines (Caco-2, SW620). As far as we know, this is the first study in this field conducted with GP extracts obtained after SSF of GP with *Phanerochaete chrysosporium* and *Trametes gibbosa*. This research is intended to contribute to the cascading use of GP, as the same sample has already been used in our other extensive research to produce enzymes, biofuels, and animal feed.

## Materials and methods

### Standards and reagents

Ethanol (analytical grade) and sodium acetate were purchased from Gram-Mol Ltd. (Zagreb, Croatia), methanol (ultra-gradient grade) from J.T. Baker (Arnhem, The Netherlands), acetonitrile (HPLC grade) from Fisher Chemical (Loughborough, UK) and glacial acetic acid from Macron Fine Chemicals (Gliwice, Poland). 2,2-Diphenyl-1-picrylhydrazyl (DPPH), 2,4,6-tris(2-pyridyl)-s-triazine (TPTZ), 2,2′-azino-bis(3-ethylbenzothiazoline-6-sulfonic acid) diammonium salt (ABTS), ammonium persulfate, catechin, epicatechin, gallocatechin gallate, epicatechin gallate, gallic acid, ellagic acid, resveratrol, syringic acid, kaempferol, *o*-coumaric acid, ferulic acid, *p*-coumaric acid, *p*-hydroxybenzoic acid, and caffeic acid were purchased from Sigma Aldrich (Saint Louis, USA). Iron(III)chloride hexahydrate was purchased from Merck (Darmstadt, Germany). Procyanidin B1 and procyanidin B2 were purchased from Extrasynthese (Genay, France). Quercetin, protocatechuic acid and vanillic acid were purchased from Acros Organics (Geel, Belgium). Hydrochloric acid was purchased from Carlo Erba Reagents GmbH (Emmendingen, Germany). Dulbecco′s Modified Eagle′s Medium (DMEM) containing glutamine and fetal bovine serum (FBS) was purchased by GIBCO (EU), whereas trypan blue and 3-(4,5-dimethylthiazol-2-yl)-2,5-diphenyltetrazolium bromide (MTT), propidium iodide (PI), and RNase A were purchased from Sigma Aldrich (MERCK, Darmstadt, Germany). Phosphate-buffered saline (PBS) was purchased from Capricorn Scientific (Ebsdorfergrund, Germany).

### Substrate and microorganisms

GP of the variety Cabernet Sauvignon (*Vitis vinifera* L.) was purchased from a local winery (Erdut, Croatia, 2016 harvest) and stored at − 20 °C before use in the experiments. Before each experiment, GP was thawed for 24 hours at + 25 °C and coarsely ground (HR 2860, Philips). The dry matter content of GP, determined using a radiation-infrared dryer HR-73, Mettler Toledo (Switzerland) using the standard method, drying temperature at 105 °C and process termination criterion (switch off 3:1 mg to 50 s weight loss) to constant mass, ranged from 91.62 to 93.52%.

Two species of white-rot fungi, *P. chrysosporium* and *T. gibbosa*, were cultivated on potato dextrose agar medium at 27 °C for 7 days and used for biological treatment of GP.

### Biological treatment of grape pomace by *Phanerochaete chrysosporium* and *Trametes gibbosa*

Fifty grams (50 g) of the prepared GP was mixed with 30 mL of distilled water in laboratory jars, autoclaved (121 °C / 20 min) and cooled overnight. In this way, the initial moisture of GP was adjusted to 60%, since the growth of white rot fungi and their metabolism under SSF conditions require a substrate humidity of 60–80%. SSF was performed to degrade the complex lignocellulosic structure of the GP to release the simple phenolic compounds.

After adjusting the moisture content of the substrate, GP was inoculated with 5 mycelial plugs of white rot fungi (diameter 1 cm) suspended in 10 mL of sterile distilled water. Experiments were performed separately for *P. chrysosporium* and *T. gibbosa* in triplicate. After inoculation, biological treatments were performed at + 27 °C for 2, 4, 10, and 15 days in an incubator with a blower set at 10% (KB 115, BINDER GmbH, Germany). The biological treatment was terminated by sterilization (121 °C / 20 min). Control samples from GP were prepared according to the same protocol but without inoculation with white rot fungi.

After treatment, all samples were dried at room temperature for 48 hours, then milled to 1 mm particle size using an ultracentrifugal mill (Retsch ZM200, Germany) and stored at + 4 °C until extraction and further analysis.

### Preparation of grape pomace extracts

Solid-liquid extraction was performed to obtain GP extracts rich in phenolic compounds. 1 gram (1 g) of the biologically treated GP was extracted using 50% aqueous ethanol as solvent with a solid/liquid ratio (S/L) of 0.025 g/mL in a water bath (SW-23, Julabo, Germany) by shaking at 200 rpm and 80 °C for 120 min. After extraction, samples were centrifuged at 10000 x *g* for 10 min (Hermle Z 326 K, HERMLE Labortechnik GmbH, Germany). Experiments were performed in duplicate. Extracts of untreated GP (control) were prepared according to the same protocol. The obtained liquid extracts were used for further analysis.

### Determination of phenolic compounds by ultra-high performance liquid chromatography

Quantification of individual phenolic compounds (flavan-3-ols, flavonols, stilbenes, and phenolic acids) in the liquid extracts of GP was performed by ultra-high performance liquid chromatography (UHPLC) according to the previously published method [26]. Photodiode array detection was performed by scanning between 252 and 370 nm. Separation was performed using a reversed phase Kinetex® C18 core-shell column (100 × 4.6 mm, 2.6 μm, Phenomenex). Analysis was performed in triplicate and results were expressed as mass of individual polyphenolic compounds per dry weight of grape pomace (mg/g_db_). The term total phenolic compound content determined by the UHPLC method (TPC) refers to the sum of the compounds quantified in this study.

### In vitro assays of the biological activities of the grape pomace extract

#### Determination of antioxidant activity (AA)

The antioxidant activity of the extracts of GP, which were biologically treated with *P. chrysosporium* and *T. gibbosa* for 4 days, was determined by DPPH, FRAP, and ABTS assays using a UV-VIS spectrophotometer (UV/VIS Spectrophotometer UV-1800, Shimadzu, Japan). The DPPH assay was performed according to the method of Bucić-Kojić et al. [27]. The FRAP assay was performed according to the method of Benzie and Strain [28] with some modifications. Briefly, the samples were prepared by mixing 2.7 mL of FRAP reagent, 270 μL of distilled water and 150 μL of extract, and the absorbance was read at 592 nm after 40 min of incubation in the dark at 37 °C. The blank sample was prepared in the same way but distilled water was used instead of extract. The ABTS assay was performed according to the method of Re et al. [29], but with modifications. Briefly, 950 μl of a diluted ABTS• + radical solution was added to 50 μl of the extracts. The absorbance was measured after 10 min of incubation in the dark at 734 nm. The control sample was prepared in the same way, but ethanol was used instead of the sample. Absolute ethanol was used as a blank. All tests were performed in triplicate, and results were expressed in Trolox equivalents per dry basis of GP (g_TROLOX_/g_db_).

#### Cell culturing

The antiproliferative effect of the extracts prepared from GP biologically treated with *P. chrysosporium* and *T. gibbosa* for 4 days was tested on two human colon carcinoma cell lines: Caco-2 (ATCC® HTB-37™) and SW-620 (ATCC® CCL-227™). Cells were cultured in DMEM supplemented with 10% FBS and 2 mM glutamine in tissue culture flasks (BD Falcon, Germany) in humidified atmosphere under the conditions of 37 °C / 5% CO_2_ gas in CO_2_ incubator (Shell Lab, Sheldon Manufacturing, USA). The trypan blue exclusion method was used to assess the viability of the cells.

#### Determination of antiproliferative activity

Extracts from GP were filtered through a 0.22 μm syringe filter and prepared in sterile deionized water at a concentration (S/L ratio) of 0.25 mg/mL, 1.00 mg/mL, 1.75 mg/mL, and 2.5 mg/mL.

The antiproliferative effect of the extracts of GP was determined by cell viability using the MTT assay [30]. Cells were seeded in 96-well flat-bottomed plates (Greiner, Frickenhausen, Austria) at a concentration of 2 × 10^4^ cells/mL and left overnight in the CO_2_ incubator to attach to the plate surface. 72 hours (72 h) after addition of the extracts from GP, the growth medium was discarded and 5 mg/mL MTT was added. After 4 hours of incubation at 37 °C, the water-insoluble MTT formazan crystals were dissolved in DMSO. The absorbance was measured at 595 nm using an Elisa microplate reader (iMark, BIO RAD, Hercules, CA, USA). Control cells were grown under the same conditions. Blank means medium without cells containing MTT. All experiments were performed at least three times in triplicate. The percentage of cell viability (CV, %) was calculated according to eq. (1):

1 $$\textsf{CV}=\frac{A_{sample}-{A}_{background}}{A_{control}-{A}_{background}} \bullet 100\left(\%\right)$$

GI_50_ value, defined as compound concentration (mg/mL) that results in a 50% reduction in cell viability, was calculated and used as a parameter for comparing the inhibitory effect between GP samples.

#### Cell cycle analysis

Cells (1 × 10^5^ cells/mL) were seeded in 6-well plates (Falcon, Durham, SAD). After 24 hours GP extracts were added at concentrations (S/L ratio) corresponding to GI_50_ (Table 1) and allowed to stand for 48 hours. Floating and adherent cells were collected separately, combined, washed with PBS fixed with 70% ethanol and stored at − 20 °C until analysis. Cell pellets were washed twice with PBS, resuspended in 1 mg/mL of PI and 0.2 mg/mL RNase A, and left in dark/cold atmosphere for 30 min. The stained cells were analysed using FACSanto II (Becton Dickinson, USA) flow cytometer (20.000 cells were analysed). The assays were performed in duplicate and repeated twice. FlowJo software, version 10.2, with the implementation of the Watson model was used to analyse the DNA histograms.

**Table 1:** GI_50_ concentration (S/L ratio) of GP extracts applied to the cell cycle of Caco-2 and SW620 for 48 hours

**Cell line**	**Treatment**^a^	**GI**_**50**_ **(mg/mL)**
Caco-2	PC	1.08
	TG	0.91
SW620	PC	0.98
	TG	1.16

^a^ TG - biological treatment by *T. gibbosa*; PC - biological treatment by *P. chrysosporium*

#### Statistical analysis

STATISTICA 13.5 (TIBCO Software Inc., Tulsa, USA) was used for statistical analysis of the results. All results were expressed as mean values. The mean values of phenolic compound content and antioxidant activity of the tested extracts were compared using Student’s t-test at a confidence level of at least 95%. Student’s t-test and non-parametric Mann-Whitney U-test were used to compare MTT data and cell data. Differences were considered statistically significant at *p* < 0.05.

## Results and discussion

### Phenolic content of biologically treated grape pomace

**Fig. 1:**
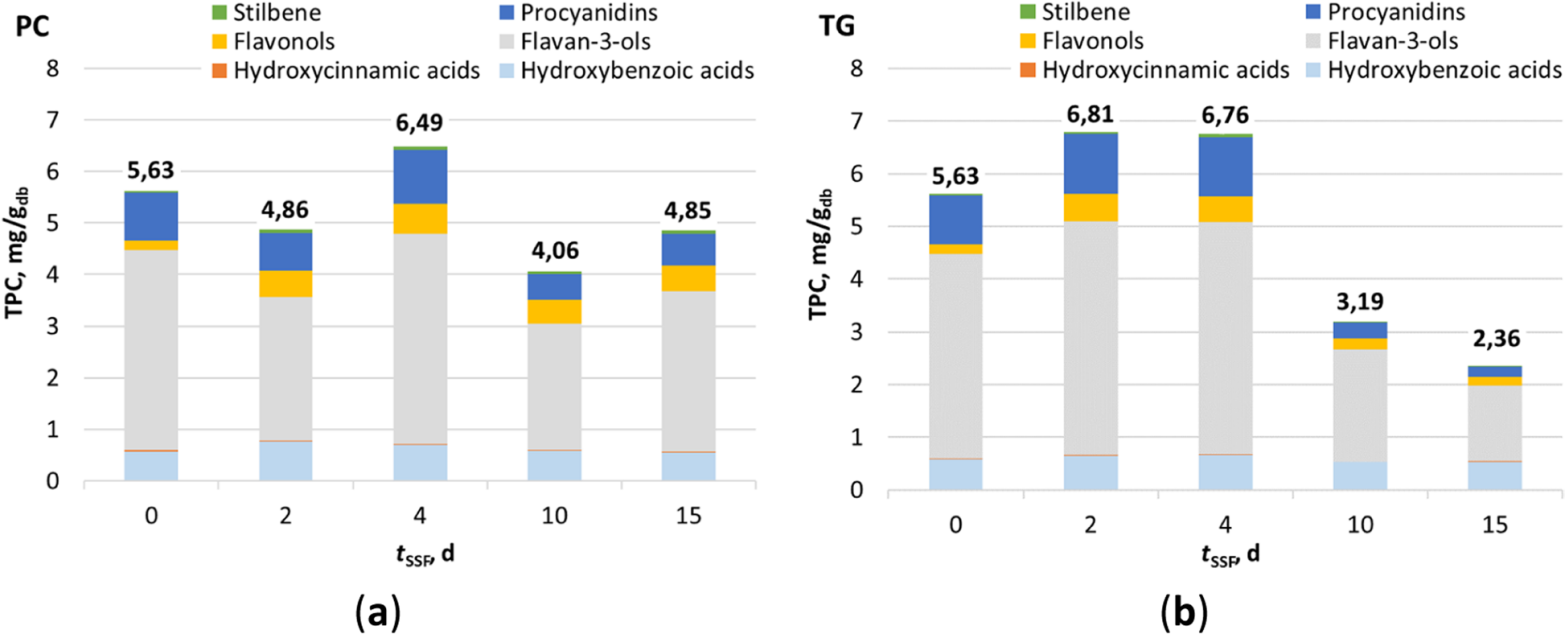
Total phenolic compound content (TPC) determined by UHPLC in extracts from GP, which where biologically treated with **a** *P. chrysosporium* (PC) and **b** *T. gibbosa* (TG) during the 15 days

The biological treatment of GP was carried out under the initial assumption that the white-rot fungi tested synthesize enzymes during their growth at GP that degrade the lignocellulose structure and release simpler phenolic compounds that are more accessible for extraction. Figure 1 shows the content of total phenolic compounds (TPC) in extracts prepared from GP biologically treated with *P. chrysosporium* and *T. gibbosa* for 15 days. Day “0” represents a control sample that was not biologically treated with white-rot fungi. Flavan-3-ols, representing the sum of the catechin, epicatechin, gallocatechin gallate, and epicatechin gallate content, were the most abundant group of phenolic compounds (57.10–68.87% of TPC) in all GP extracts tested, followed by hydroxybenzoic acids (10.19–22.74% of TPC), procyanidins (8.31–16.84% of TPC) and flavonols (3.26–11.60% of TPC). The hydroxybenzoic acids include the sum of the contents of gallic acid, ellagic acid, syringic acid, protocatechuic acid, vanillic acid, and *p*-hydroxybenzoic acid. Procyanidins comprise the sum of the content of the oligomers procyanidin B1, and procyanidin B2 and flavonols represent the sum of the content of kaempferol and quercetin. Resveratrol (0.81–1.33% of TPC), representing a group of stilbenes, and hydroxycinnamic acids (0.19–0.43% of TPC), comprising the sum of the contents of ferulic acid, *o*-coumaric acid, *p*-coumaric acid, and caffeic acid, were determined in small amounts and with smaller contributions to TPC than the above phenolic compounds.

**Fig. 2:**
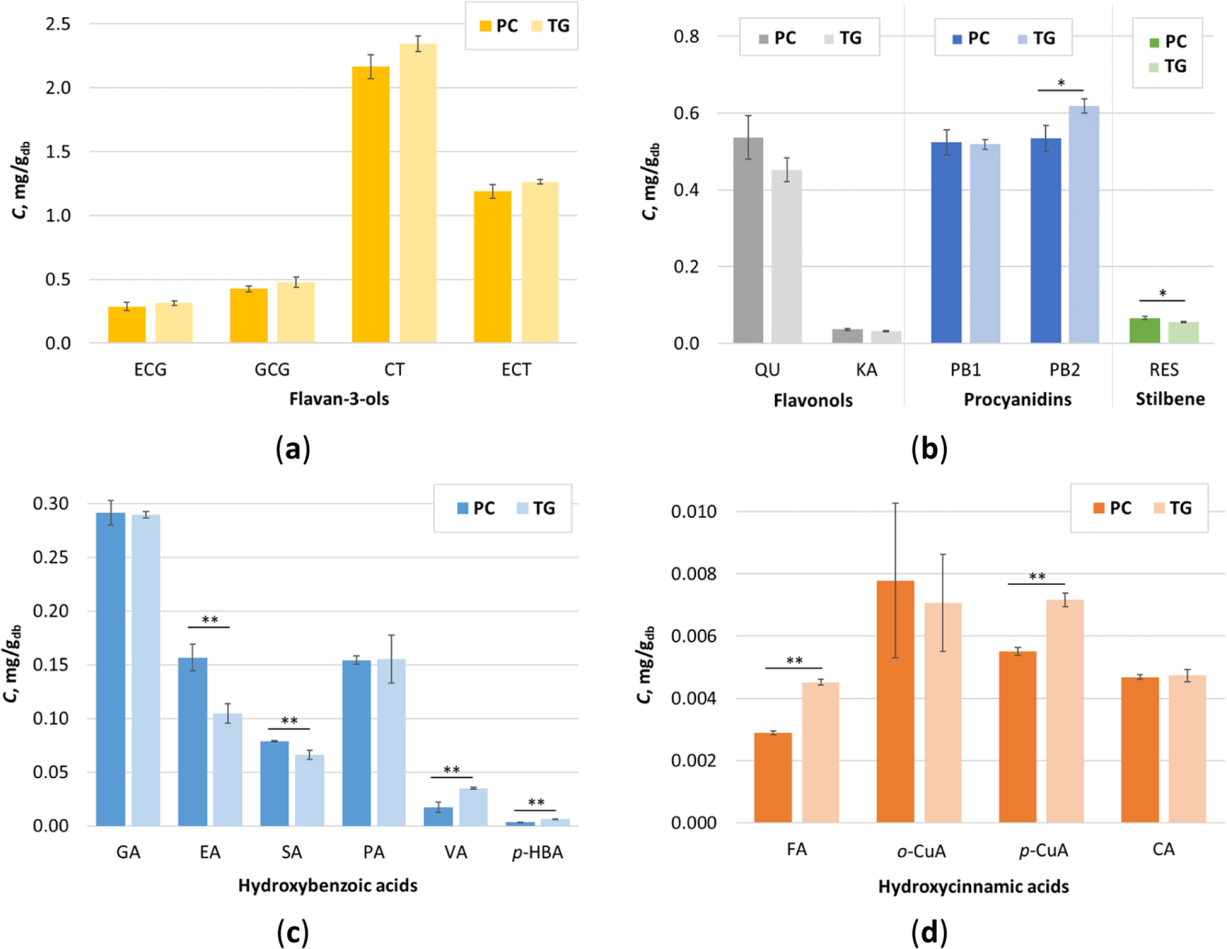
Content of individual phenolic compounds (*C*) determined by UHPLC in extracts obtained from biologically treated GP by *P. chrysosporium* (PC) and *T. gibbosa* (TG) after 4 days of fermentation: **a** flavan-3-ols; **b** flavonols; **c** hydroxybenzoic acids and **d** hydroxycinnamic acids. Error bars correspond to SD. (*) - statistically significant difference between 95% extracts at confidence level (Student’s t-test); (**) - statistically significant difference between extracts at 99% confidence level (Student’s t-test)

In both cases (Figure 1), it can be seen that SSF with white-rot fungi had a positive effect on the release of phenolic compounds and their extractability. In the case of *P. chrysosporium*, the highest TPC (6.49 mg/g_db_) was reached after 4 days of fermentation, while in the case of *T. gibbosa*, the highest TPC was reached after 2 days (6.81 mg/g_db_) of biological treatment. Compared to the control sample (5.63 mg/g_db_), the observed increase in TPC was statistically significant (*p* < 0.05). It was also found that there was no statistically significant (*p* < 0.05) difference between the TPC of the samples treated with *T. gibbosa* for 2 and 4 days. Therefore, the GP samples treated with *P. chrysosporium* and *T. gibbosa* for 4 days under SSF conditions were selected for further studies to determine the biological activity (antioxidant and antiproliferative activities) of the GP extracts. 19 individual phenolic compounds were identified in the extracts obtained from GP after the fourth day of biological treatment by *P. chrysosporium* and *T. gibbosa* (Fig. 2).

Catechin was the dominant single phenolic compound in both extracts (2.17 mg/g_db_ for *P. chrysosporium* and 2.34 mg/g_db_ for *T. gibbosa*) followed by epicatechin (1.19 mg/g_db_ for *P. chrysosporium* and 1.26 mg/g_db_ for *T. gibbosa*) (Fig. 2a). These values are higher than those published by Negro et al. [31] for the biologically untreated GP variety Cabernet Sauvignon. In our study, other compounds were detected at lower concentrations and generally did not follow the same trend in both extracts (Fig. 2a-d). This can be explained by the specificity of each SSF process, which depends on a number of factors, but mainly on the microorganism and the properties of the substrate used. Statistical analysis (Student’s t-test) showed that there was no statistically significant difference between the determined contents of catechin, epicatechin, epicatechin gallate, gallocatechin gallate, quercetin, kaempferol, procyanidin B1, gallic acid, protocatechuic acid *o*-coumaric acid, and caffeic acid in both extracts. On the other hand, the content of procyanidin B2 and resveratrol in the extracts from GP treated with *P. chrysosporium* and *T. gibbosa* was statistically significant at 95% confidence level, and the content of ellagic acid, syringic acid, vanillic acid, *p*-hydroxybenzoic acid, ferulic acid, and *p*-coumaric acid was statistically significant at 99% confidence level (Fig. 2a-d).

### Antioxidant activity of biologically treated grape pomace extracts

**Fig. 3:**
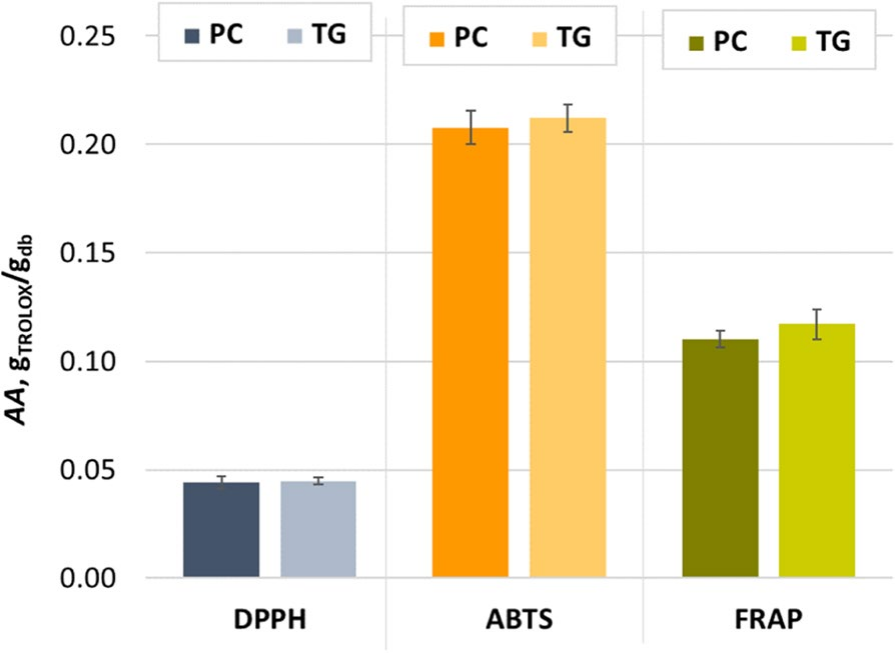
Antioxidant activities (*AA*) of extracts from GP after the fourth day of biological treatment by *P. chrysosporium* (PC) and *T. gibbosa* (TG) measured by DPPH, ABTS, and FRAP methods. Histogram bars represent the mean of six measurements and error bars represent SD

Phenolic compounds contribute to antioxidant activity and have a positive effect on human health by reducing the risk of developing chronic diseases such as cancer, diabetes, inflammatory and cardiovascular diseases. The antioxidant activity of phenolic compounds depends on their degree of hydroxylation and conjugation [32]. The antioxidant activity evaluated by the three assays (DPPH, ABTS, and FRAP) showed a similar trend for the extracts obtained after the fourth day of biological treatment of GP by *P. chrysosporium* and *T. gibbosa*, although it can be seen in Fig. 3 that the extracts obtained after SSF with PC showed a slightly lower *AA*. Student’s t-test showed that there is no statistically significant difference of *AA* between the tested extracts at 95% and 99% confidence level for the same assay. Dulf et al. [33] found an increase in antioxidant activity when GP was biologically treated with the filamentous fungi *Actinomucor elegans* (21.42% after 4 days SSF) and *Umbelopsis isabellina* (16% after 8 days SSF). Martínez-Ávila et al. [34] found that the SSF of GP with *Aspergillus niger* could improve the antioxidant activity of GP extracts by 5 to 20% during 18 hours. They observed that the decrease in antioxidant activity after 18 hours of biological treatment coincided with a decrease in gallic acid content. Leite et al. [35] showed that the antioxidant activity of extracts from winemaking residues (exhausted grape marc and vine trimming shoots) was most affected by SSF with *Rhizopus oryzae* among all filamentous fungi tested, while SSF with *Aspergillus niger* resulted in the highest antioxidant activity of extracts from grape stalks. Certain phenolic compounds can be bound to the structure of hemicellulose and lignin, and the action of fungal enzymes during SSF can influence the release of free phenolic compounds and consequently the increase of antioxidant activity [35]. Antioxidant activity can be correlated with the phenolic content of the extract and often results from the synergistic action of active substances. Therefore, the phenolic profile of the extract determines its efficacy, as the individual activity of the represented substances can vary greatly [36]. Since phenolic compounds have high antioxidant activity and their content changes during biological treatment, this also affects the radical scavenging properties of the fermented GP. The fact that antioxidants affect numerous important signaling pathways and targets associated with antiproliferative effects is well recognized, and numerous studies have been devoted to investigating the utility of certain bioactive compounds and their effectiveness as inhibitors of colon cancer cell growth [37, 38].

### Antiproliferative effect of biologically treated grape pomace extracts

**Fig. 4:**
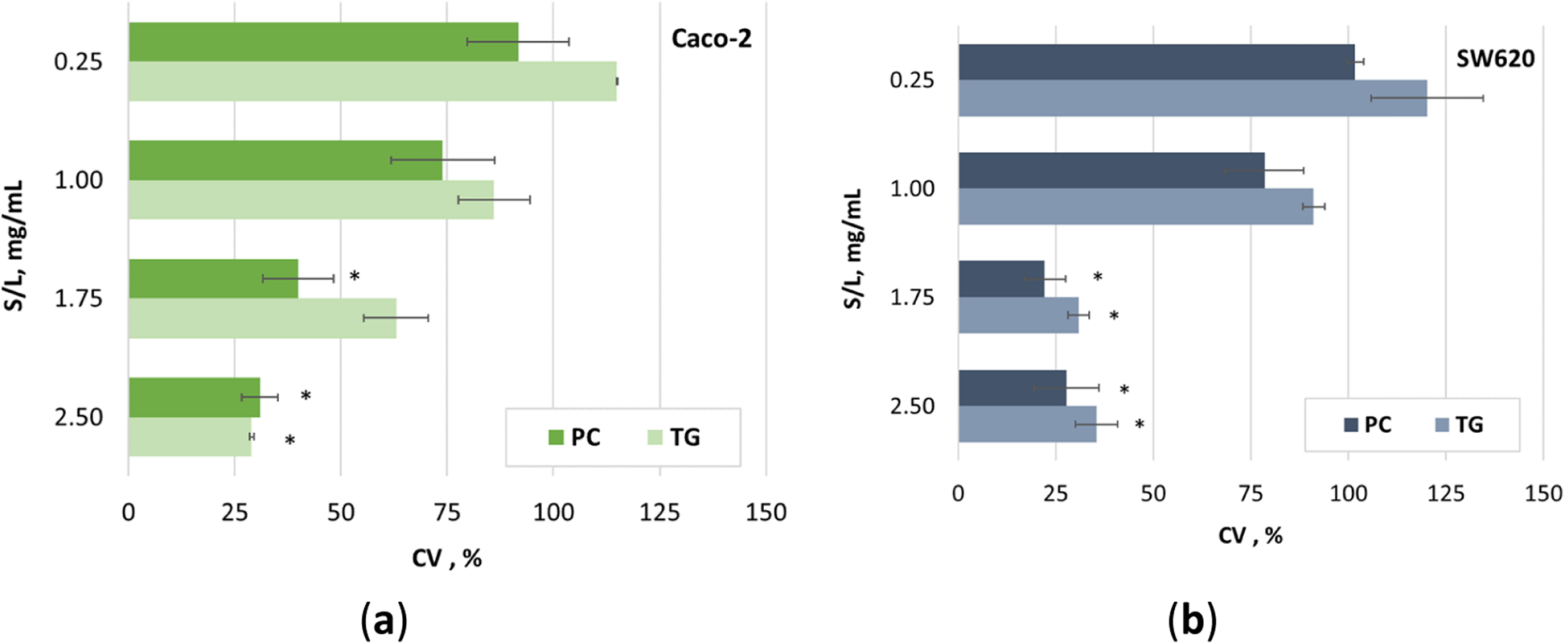
Antiproliferative effect of extracts from GP after the fourth day of biological treatment by *P. chrysosporium* (PC) and *T. gibbosa* (TG) on: **a** Caco-2 and **b** SW620 cell line. Data are presented as percentage of cell viability (CV, %) as a function of concentration of the two different extracts. Histogram bars represent the mean of three independent experiments performed in triplicate, and error bars correspond to SD. Values marked with (*) are statistically significant (*p* < 0.05) with respect to the control group, as determined by the Mann-Whitney U-test

Phenolic compounds such as catechin, resveratrol, gallic acid, etc. are known to be radical scavengers, have antiproliferative effects on tumors, and protect membranes from oxidation. Numerous studies have demonstrated the key role of cancer therapy in identifying compounds that induce cell apoptosis and play a key role in eliminating mutated hyperproliferating cells from the system. Phenolic compounds, with their antiproliferative and antitumor activities, are involved in the proliferation, differentiation, and apoptosis of various cancer cells, including colon cancer [4, 39]. Also very important in terms of chemoprevention by phenolic compounds is their differential effect on cancer cells but not on normal cells [40]. In this study, the inhibitory effect of GP extracts on cell growth of two colon cancer cell lines was investigated. The results showed a difference in efficacy in cell growth regression between Caco-2 and SW620 cells depending on the concentration of the extract and the biological treatment (Fig. 4). Both types of colon cancer cell lines were more sensitive to the presence of extracts prepared from *P. chrysosporium*-treated GP than to extracts prepared from *T. gibbosa*-treated GP at the same concentration (S/L ratio), except at the highest concentration (2.5 mg/mL), where a slightly lower cell viability of Caco-2 cells was observed in the presence of extracts prepared from *T. gibossa*-treated GP. An inhibitory effect on Caco-2 cell growth was observed for GP treated with *P. chrysosporium* at the two highest concentrations (S/L ratio), 1.75 and 2.5 mg/mL, resulting in a 60.1 and 69.1% reduction in cell growth, respectively. On the other hand, the greatest effect on reducing Caco-2 cell viability was observed when Caco-2 cells were treated with *T. gibbosa*-treated extract GP at a concentration of 2.5 mg/mL, resulting in an inhibitory effect of 71.0%. Compared to the control sample, these three effects were statistically significant (*p* < 0.05) (Fig. 4a). Martins et al. [22] studied the antiproliferative effect of biotransformed grape pomace by tannase on Caco-2 cells and their results are less striking than ours, since they studied a lower concentration (100–500 μg/mL). Dükel et al. [41] investigated the antiproliferative effects of flavonoids (catechin, epicatechin, and naringenin) in colon cancer cells (Caco-2, DLD-1, and SW620) and normal colon epithelial cells (CCD18Co) and found that all flavonoids showed cytotoxic effects on all cell lines studied at a treatment of less than 25 μM according to IC_50_ values. In addition, all flavonoids used showed high cytotoxicity against the Caco-2 adenocarcinoma cell line at low concentrations, while naringenin inhibited proliferation in the SW620 metastatic cell line at low concentration (IC_50_ 11 ± 1.9 μM). The study conducted by Parry et al. [21] investigated the cytotoxic activity of GP on Caco-2 without biotransformation. Their results suggest a less pronounced antiproliferative activity compared with our results. Possible reasons for the different results of the studies dealing with the antiproliferative effect on the Caco-2 cell line are the different geographical origin of the grape pomace, the manufacturing process, and the presence of a biotransformation step. Another possible reason for the different results is the form of the grape pomace extract studied. We tested liquid extracts without prior lyophilisation. All the previously mentioned reasons related to Caco-2 cells and GP origin are related to the cytotoxic results obtained for the extracts of GP on the SW620 cell line. However, the results are somewhat different compared to Caco-2 cells [21, 22]. Specifically, higher concentrations (S/L ratio) of both types of extracts (1.75 mg/mL and 2.5 mg/mL) showed a statistically significant (*p* < 0.05) inhibitory effect on SW620 cell growth, while lower concentrations (0.25 mg/mL; 1.00 mg/mL) had no effect on SW620 cell line growth (Fig. 4b). The extract of GP, treated with *P. chrysosporium*, had the greatest inhibitory effect at a concentration of 1.75 mg/mL (77.9%), followed by the same type of extract at a concentration of 2.5 mg/mL (72.3%), followed by GP extracts after treatment with *T. gibbosa* at a concentration of 1.75 mg/mL (69.1%) and 2.5 mg/mL (64.6%). Using the results of the antiproliferative assay, the growth inhibitory concentration (S/L ratio) leading to a 50% reduction in cell growth is calculated (GI_50_). These values were used to evaluate the effects of the extracts of GP on the cell cycle of Caco-2 and SW620 cells (Table 1).

Further evaluation of the tested GP extracts in GI_50_ concentration (S/L ratio) showed an outstanding effect on SW620 and Caco-2 cell cycle after 48 hours of exposure. Both extracts cause different changes in cell cycle ratio compared to control and each other. GP treated with *T. gibbosa* reduces the percentage of SW620 cells in S phase by 9.8% and increases the percentage of cells in G2/M phase by 6.8% compared to control. GP treated with *P. chrysosporium* induces the change in G1 cell phase and increases the percentage of cells by 7.7% without significant change in the percentage of cells in S and G2/M phase (Table 2).

**Table 2:** Cell cycle phases (G1, S, G2/M) of Caco-2 and SW620 cell lines (mean (SD))

**Cell line**	**Treatment**^**a**^	**G1 (%)**	**S (%)**	**G2/M (%)**
Caco-2	Control	55.9 (0.4)	30.1 (0.4)	9.4 (0.2)
	PC	58.3 (0.6)	23.3 (2.2)	11.7 (2.9)
	TG	49.1 (0.6)	34.0 (0.1)	8.8 (0.4)
SW620	Control	50.1 (0.7)	26.7 (0.3)	12.7 (0.2)
	PC	57.8 (0.4)*	20.9 (1.7)	13.4 (0.1)
	TG	54.7 (0.6)	16.9 (0.9)*	19.5 (0.9)*

^a^ PC - biological treatment by *P. chrysosporium*; TG - biological treatment by *T. gibbosa*. * Statistically significant at confidence level at 95% (Student’s t-test)

The effect of GP, treated with *P. chrysosporium*, on the cell cycle of Caco-2 was complementary to the effect of SW620 but less pronounced. GP, which was treated with *T. gibbosa*, had a different effect on the cell cycle of Caco-2 cells than SW620 cells. The percentage of cells in G1 and G2/M phase was slightly reduced, while S phase was increased by 3.9% compared with control (Table 2). The difference between the total number of cells (100%) and the cells in the cell phases (4.6 to 10.5%) is probably due to the Watson model used, which does not include apoptotic or dead cells in the presentation of the results. Ho et al. [42] investigated the antiproliferative effect of *Barringtonia racemosa* leaf water extract (BLE) and gallic acid (GA) on Caco-2 cells for 48 h. For cell treatment, they used IC_20_ (69.1 μg/ml) and IC_50_ (325.5 μg/ml) concentrations of BLE and IC_20_ (3.7 μg/ml) and IC_50_ (10.6 μg/ml) concentrations of gallic acid. All treatments significantly decreased the percentage of cells in G0/G1 phase compared with control (Caco-2 cells grown only in MEM). Meanwhile, only BLE IC_50_ and GA IC_50_ caused a significant increase in the percentage of cells in S phase, and only cells treated with gallic acid had a significantly higher percentage of cells in G2/M phase. The cell cycle of mammalian cells is tightly controlled by several checkpoints and protein complexes formed by cyclins and cyclin dependent kinases. The cell cycle goes through four phases in a uniform order from G1 to S, followed by G2 and M [43], from which two new cells emerge. While most cells in the human body do not enter division until necessary, malignant cells divide rapidly because they lack the key control mechanisms of division. As a result, they proliferate rapidly and uncontrollably. Polyphenolic compounds affect the cell cycle through four possible mechanisms: antioxidant activity, regulation of p53 protein, inhibition of protein kinase activity [44], modulation of cyclin, cyclin dependent kinases or anaphase promoting complex or cyclosome (APC/C), which arrest the cell cycle [45], and apoptosis [4]. It has been reported that certain phenolic compounds cause cell cycle changes or cell arrest in cancer cells. For example, in colorectal cancer cells, quercetin causes cell cycle arrest in S phase and a reduction in the proportion of cells in G0/G1 phase (LoVo49) [46], whereas kaempferol causes cell cycle arrest in G1 and G2/M phase [47]. Rutin has also been reported to induce G2/M arrest in human neuroblastoma cells, LAN-5 [48]. Epigalocatechin inhibits the activity of cyclin-dependent kinases by inducing the expression of inhibitors of cyclin-dependent kinases p21 and p27 [29]. Resveratrol, one of the most abundant grape polyphenols, has the potential to stop colon cancer cell growth [2]. It inhibits tumour cell proliferation by stopping cell growth at different stages of the cell cycle depending on the cell line [49]. In malignant colorectal cancer cells, resveratrol induces apoptosis through activation of proapoptotic p53 and activation of caspase 3 and 8 through production of reactive oxygen species [50]. A study by Schneider et al. [51] showed that resveratrol generally has an inhibitory effect on cell growth of Caco-2 cells, with no toxic effect stopping cells in the G2/M phase of the cell cycle. Our results related to the cell cycle of SW620 cells exposed to an extract of GP treated with *T. gibbosa* are consistent with results indicating an increased number of cells in the G2/M phase. Although the concentration of resveratrol in our samples is not very high (0.07 mg/g_db_ for GP-treated with *P. chrysosporium* and 0.06 mg/g_db_ for GP-treated with *T. gibbosa*), other components such as catechin, epicatechin, procyanidin B1 and B2 are present in significant amounts. Considering the different effects of each polyphenolic compound on the cell cycle, it is likely that a synergistic effect occurs when these compounds are included as a mixture in the extracts used. Quercetin is another possible cause of cell cycle arrest of SW620 cells in G2/M phase. Quercetin disrupts the link between beta-catenin and transcription factor 4 while reducing the expression of survivin and cyclin D [50]. Quercetin in the extract prepared from GP treated with *T. gibbosa* and *P. chrysosporium*, is present at a concentration of 0.45 mg/g_db_ and 0.54 mg/g_db_, respectively, indicating a possible cumulative effect on the antiproliferative activity and cell cycle impairment of Caco-2 and SW620 cell lines. Our data on the cell cycle of SW620 and Caco-2 cells suggest that there is no clear biological trend of effect between the samples studied treated with *P. chrysosporium* and *T. gibbosa* microorganisms. These discrepancies are likely the result of biological treatment with *P. chrysosporium* and *T. gibbosa* fungi.

## Conclusions

The studied grape pomace extracts from biologically treated Cabernet Sauvignon are rich in phenolic compounds such as catechin, epicatechin, procyanidin B1, procyanidin B2 and quercetin. Most of the health-promoting effects of phenolic compounds are related to their antioxidant capabilities. By combining various mechanisms, including antioxidant activity, antiproliferation effect, cell cycle inhibition, induction of apoptosis, etc., they may play an important role in cancer prevention. The evaluation of the biological activity of grape pomace extracts against Caco-2 and SW620 cell lines suggests an antiproliferative activity and a reduction in cell growth of these two colorectal cancer cell lines. The studied grape pomace extracts induce changes in the cell cycle of the SW620 cell line, but the differences in the observed effect indicate a different mode of action, probably caused by fermentation in the presence of different fungi, i.e. *P. chrysosporium* and *T. gibbosa*. The obtained results suggest the presence of bioactive compounds in biotransformed grape pomace as a residue from winemaking, which could be used to prevent colon cancer. Future research will aim to determine the sequence of cascading utilization of biotransformed grape pomace to maximize its use for the production of high-value products, including phenolic-rich extracts, while approaching the zero-waste concept.

## Data Availability

The datasets used and/or analysed during the current study are available from the corresponding author on reasonable request.

## Abbreviations

AA: antioxidant activity
ABTS: azino-bis(3-ethylbenzothiazoline-6-sulfonic acid) diammonium salt
CA: caffeic acid
Caco-2: colorectal adenocarcinoma cells
CT: catechin
CV: cell viability
DMEM: Dulbecco’s Modified Eagle’s Medium
DMSO: dimethyl sulfoxide
DPPH: 2,2-diphenyl-1-picrylhydrazyl
EA: cell viability
ECG: epicatechin gallate
ECT: epicatechin
FA: ferulic acid
FBS: fetal bovine serum
GA: gallic acid
GCG: gallocatechin gallate
GI_50_: 50% reduction in cell growth
GP: grape pomace
KA: kaempferol
MTT: 3-(4,5-dimethylthiazol-2-yl)-2,5-diphenyltetrazolium bromide
*o*-CuA: *o*-coumaric acid
PA: protocatechuic acid
PB1: procyanidin B1
PB2: procyanidin B2
PBS: phosphate buffered saline
PC: *Phanerochaete chrysosporium*
*p*-CuA: *p*-coumaric acid
*p*-HBA: *p-*hydroxybenzoic acid
PI: propidium iodide
QU: quercetin
RES: resveratrol
S/L: solid/liquid ratio, concentration (mg/mL)
SA: syringic acid
SD: standard deviation
SSF: solid-state fermentation
SW620: colon adenocarcinoma cells
TPTZ: 2,4,6-tris(2-pyridyl)-s-triazine
TG: *Trametes gibbosa*
TPC: total phenolic compounds
UHPLC: ultra-high performance liquid chromatography
VA: vanillic acid
